# Repeat At-Home Saliva Collection to Measure Cortisol Awakening Response (CAR): Protocol Feasibility Study Among Black Women With Histories of Abuse

**DOI:** 10.2196/64150

**Published:** 2026-09-03

**Authors:** Kiyomi Tsuyuki, Marguerite B Lucea, Andrea N Cimino, Michael Killian, Christina J Catabay, Engle Abrams, Emmanuel Baffour-Siaw, Jacquelyn C Campbell, Douglas A Granger, Jamila K Stockman

**Affiliations:** 1 Division of Infectious Diseases and Global Public Health, Department of Medicine, University of California, San Diego, 9500 Gilman Drive, Mail Code 0507, La Jolla, CA, United States; 2 Department of Nursing, College of Health Professions, Towson University, Towson, MD, United States; 3 Johns Hopkins University School of Nursing, Baltimore, MD, United States; 4 College of Social Work, Florida State University, Tallahassee, FL, United States; 5 Department of Psychology, University of California at Irvine, Irvine, CA, United States; 6 Department of Pediatrics, Johns Hopkins University School of Medicine, Baltimore, MD, United States

**Keywords:** saliva collection, black women, histories of abuse, gender-based violence, cortisol, stress biomarkers, immune dysregulation, protocol adherence

## Abstract

**Background:**

Emerging evidence supports that women with histories of abuse have heightened stress and immune dysregulation. Few studies have examined the biological plausibility of this association in US Black women—a population disproportionately affected by gender-based violence (GBV), health disparities, and generally underrepresented in research. Biomarkers of stress and immune health remain challenging to study due to barriers in recruitment, retention, and protocol adherence.

**Objective:**

This study developed and examined the feasibility of an at-home, self-collected, and minimally invasive salivary cortisol awakening response (CAR) collection protocol among Black women with histories of abuse in Baltimore City, Maryland.

**Methods:**

Black women were recruited from November 2015 to May 2018 from Baltimore City sexually transmitted disease clinics. Participants received in-person instruction and demonstration on saliva self-collection using the passive drool method, and were provided study-issued cell phones for reminders and adherence tracking. Participants self-collected saliva samples upon waking and 30 minutes post waking on 3 consecutive days to assess CAR. Outcomes included protocol completion, self-reported adherence, and experiences (via saliva collection logs), a weighted protocol adherence score, and cortisol measures (waking, post waking, and CAR). Differences in sociodemographic characteristics and outcome measures were assessed by adult forced sex exposure status using chi-square tests and ANOVA. The Pearson correlation coefficient assessed the day-to-day reproducibility of cortisol measures among women with 2 full days of valid samples.

**Results:**

Of the 305 women completing the study survey, 228 completed the saliva specimen collection protocol, with no significant differences in completion between women with and without forced sex histories. Protocol feasibility was high, with 84% (191/228) completing at least 1 full day of adherent saliva collection and more than half (132/228) completing 2 full days. While feasibility did not differ by exposure status, women with adult forced sex exposure demonstrated lower protocol adherence scores compared to their unexposed counterparts. Between-day cortisol measures demonstrated variable reproducibility across waking, post waking, and CAR measures.

**Conclusions:**

This at-home salivary cortisol collection protocol was feasible in our robust sample of Black women with histories of abuse. The process of at-home collection of salivary biomarkers, including cortisol measures, was well-documented, and participants were able to adhere to it. The protocol yielded usable data that will facilitate the examination of the physiological and health repercussions of GBV.

## Introduction

### Background

Chronic social stress is increasingly recognized as a key contributor to health disparities [1]. The minority stress model posits that individuals exposed to intersecting forms of social disadvantage experience cumulative stress that can be biologically embedded over time and adversely affect health [2]. Black women may face particularly high levels of chronic stress due to the intersection of racism, sexism, and a disproportionate burden of gender-based violence (GBV) [3,4]. Despite these intersecting stressors, few studies have incorporated biological measures of stress in research with women exposed to GBV. Existing studies often rely on small sample sizes, lack racial and ethnic diversity, collect clinic-based biomarkers, limiting participation and generalizability to the social context, and lack measures of collection protocol adherence [5]. Developing and testing a feasible and acceptable trauma-informed protocol for at-home, self-collection of stress biomarkers is a critical step toward advancing health equity research on the physiological impacts of violence. In this paper, we establish a protocol of repeat salivary cortisol self-collection among a robust sample of Black women with experiences of violence and examine the feasibility and usability of the samples.

GBV is a pervasive public health problem. Globally, an estimated 30%‐60% of women experience physical and/or sexual violence during their lifetime [6]. In the United States, 42% of women experience physical intimate partner violence (IPV) and 20% experience sexual violence in their lifetime [7]. Black women experience disproportionately high rates of IPV, including physical, sexual, and stalking IPV, compared with their White and Latina counterparts [7]. The health consequences of GBV are substantial. Survivors of IPV are more likely to experience mental health conditions (eg, depression, anxiety, posttraumatic stress disorder, and suicidality) [7,8] and chronic physical conditions, including cardiovascular disease, chronic pain, gastrointestinal disorders, sexual and reproductive health disorders, and increased susceptibility to infectious diseases, than women without IPV experiences [9]. Population-based research further suggests that Black women survivors of severe IPV demonstrate higher rates of mental health disorders and report poorer overall health than their White counterparts [10].

Cortisol is a widely used biomarker of physiological stress. Researchers have measured cortisol levels among women with histories of abuse using plasma [11,12], hair [13], and saliva [11,14,15]. However, this research has yielded inconsistent findings associated with cortisol levels in the context of IPV, likely due to variation in measurement approaches and cortisol analysis [13]. Salivary cortisol has emerged as a valuable tool because it is minimally invasive, convenient, and well-suited for repeat sampling. Saliva collection enables participants to self-collect samples in naturalistic settings (eg, at home), facilitating the study of hypothalamic-pituitary-adrenal (HPA) axis activity within the context of their daily life rather than in clinical settings. This approach further supports the inclusion of larger and more diverse samples, including populations that may face barriers to participation in clinic-based research. However, salivary cortisol research among women with abuse histories has been limited by several methodological challenges, including insufficient attention to collection adherence measures, lack of racially and ethnically diverse samples, reliance on clinic-based collection, and inadequate sampling frequency within or across days, which is critical to accurately quantify cortisol levels and patterns [16].

Cortisol follows a diurnal rhythm, including a rapid increase within 30‐45 minutes after waking, known as the cortisol awakening response (CAR) [17]. Alterations in the CAR have been linked to chronic stress exposure and a range of adverse health outcomes disparities [18,19], making it a critical index in stress research. Accurate assessment of CAR depends on precise timing of saliva collection immediately upon waking and at specific postwaking intervals [20,21], introducing challenges for at-home saliva self-collection protocol adherence [22]. Although previous work has attempted to standardize CAR measurement, issues of protocol adherence remain a significant concern, particularly in unsupervised settings. These challenges may be amplified in research among women with histories of abuse, for whom ethical, safety, and trauma-informed considerations are critical. No standardized protocol exists for at-home salivary CAR self-collection for women with histories of abuse.

Medical mistrust is a critical barrier to participation in research for women with abuse histories and among Black women, and must be addressed with trauma-informed practices. Women with abuse histories often distrust medical institutions due to previous negative experiences, fear of judgment, and concerns about confidentiality [23,24]. Gender and abuse biases within medical settings can lead to the dismissal of women’s health concerns, while systemic failures in addressing abuse further exacerbate mistrust and discourage care-seeking. Among Black women, medical mistrust is rooted in both historical and ongoing discrimination within the health care system [25,26]. Black women have been disproportionately subjected to unethical medical practices, such as forced sterilization and nonconsensual experimentation, and report higher levels of perceived discrimination in health care settings [25]. Medical mistrust is linked to poorer health outcomes, contributes to health disparities, and likely reduces willingness to participate in research and specimen collection [27-30]. Addressing these barriers requires the implementation of trauma-informed care approaches. Developing an at-home, repeated measures saliva collection protocol requires attending to these barriers to ensure inclusive participation.

### Objectives

The objectives of this study are 2-fold. First, we describe the at-home, salivary CAR self-collection protocol designed for Black women with histories of abuse. Second, we assess the feasibility of this protocol among Black women with histories of abuse.

## Methods

### Study Design

Data were drawn from the ESSENCE (Examining Stress, Sexual Experiences, and Neighborhood Correlates of HIV Risk among Black Women) project, a retrospective cohort study to examine how experiences of forced sex and physiological stress response contribute to HIV risk among Black women. This study focuses on the development and implementation of an at-home saliva CAR self-collection protocol embedded within the broader study.

### Study Setting

The study was conducted in 2 publicly funded sexually transmitted disease (STD) clinics in Baltimore, Maryland, United States, from November 2015 to May 2018. Participants were recruited from neighborhoods that have historically experienced structural inequities, such as racism, residential segregation, and community disinvestment [31], as well as unethical research practices surrounding Henrietta Lacks that spurred medical mistrust in the communities [32]. Data collection occurred during a period of heightened community tension following the death of Freddie Gray in police custody in April 2015 and the subsequent criminal proceedings that ultimately exonerated the 6 officers who were involved in July 2016, and occurred in neighborhoods where the protests were most intense [33]. These contextual factors informed the study’s trauma-informed and community-engaged approach to participant recruitment and saliva collection protocol development.

### Recruitment and Eligibility

Black women seeking health services were recruited in the clinic waiting rooms by trained female research staff. To prioritize safety and confidentiality, staff discreetly approached women and invited them to participate in an anonymous study about “women’s health and safety.” Interested women were led to a private examination room where staff read the informed consent document.

After providing informed consent, participants responded to a short eligibility screening survey. Eligibility criteria included (1) biologically female, (2) between ages 18‐44 years, (3) self-identified as African American or Black, (4) reported having sex with a man in the past 6 months, and (5) reported having either 2 or more sexual partners in the past year or having a sexual partner at high risk for HIV (ie, used injection or noninjection drugs, had sex with men, been to prison, had a concurrent sex partner, had an STD, was HIV-positive, or did not know if their sexual partner had any of these characteristics).

### Ethical Considerations

The study protocol was approved by the institutional review boards of Johns Hopkins University and the University of California, San Diego. Participants provided written informed consent separately for the eligibility screening and study participation, including the survey and saliva collection components. Although the study collected geographic data and contact information for follow-up, confidentiality was maintained by storing consent forms and identifiable information separately from the survey responses and biomarker data. All study data were deidentified and linked to a participant ID. Participants received financial compensation totaling US $70 (US $10 for screening, US $25 for survey completion, and US $35 for returning saliva samples), a daily bus pass, and a list of community resources related to violence and health care service referrals.

### Survey Data Collection

Participants completed a 60‐ to 90-minute survey administered using audio computer-assisted self-interview software. The survey assessed sociodemographic characteristics, sexual health and behavior, violence and trauma history, mental health, substance use, and neighborhood and social environmental factors. A female research staff member was made available to the participants in case of any questions, concerns, or technical difficulties. Following survey completion, participants were trained on saliva collection procedures [34].

### Salivary Cortisol Collection Protocol

#### In-Clinic Training and Demonstration

##### Overview

After completing the survey, participants received standardized training on saliva collection procedures from research staff using a scripted protocol. Training covered the passive drool technique, saliva collection timing to measure the CAR, adherence documentation procedures, sample storage, and sample retrieval process. Participants then observed a demonstration of the passive drool technique and were given the opportunity to practice. Participants then received a collection kit containing 6 straws, 6 barcoded cryovials (with color-coded caps to visually distinguish waking [green] and 30-minute postwaking [red] samples), a saliva collection log (with adherence instructions on the back), a pen, and a study-issued mobile phone with charger. Study staff used the mobile phones to maintain contact with participants during the collection period to provide reminders, troubleshoot issues, and coordinate sample retrieval.

##### Passive Drool Technique

Saliva samples were collected using the passive drool method developed by Salimetrics [34]. This method collects whole saliva rather than gland-specific saliva obtained from oral swabs. Participants generated saliva by thinking about a favorite food and generating saliva by gently moving their jaws as if chewing and allowing saliva to pool in the mouth. Using a straw attached to a cryovial, participants then gently allowed the saliva to flow into the vial until the 1 mL fill-line was reached.

##### CAR Collection

Participants were instructed to collect 2 saliva samples per day for 3 consecutive days (6 samples in total)—one sample immediately upon waking and a second sample 30 minutes post waking [35]. This schedule allowed the measurement of the CAR. Participants were instructed to avoid brushing their teeth, smoking, or eating before completing each morning’s saliva collection.

##### Adherence Monitoring

The saliva collection protocol used multiple adherence monitoring strategies to measure the timing of saliva collection, as the accurate measurement of CAR requires precise timing of saliva collection relative to waking time. Participants were asked to record collection times using (1) a saliva collection log where wake times and saliva collection times were written in the log (Multimedia Appendix 1), (2) time-stamped photos of each saliva collection cryovial using the study-issued phone, and (3) text message responses to automated SMS text message prompts confirming sample collection times.

The saliva collection log included saliva protocol instructions, vial ID numbers, and space to document any stressors or other issues that may have impacted saliva collection. Study staff recorded participants’ anticipated wake times and expected collection times for each study day on the saliva collection log. In the saliva collection log, participants were further instructed to report any mistakes or protocol deviations during saliva collection, as well as stressors experienced the previous night.

SMS text message reminders were delivered via an automated SMS digital platform and were scheduled based on participants’ anticipated wake times. Messages prompted participants to confirm when they woke up and when they began collecting both the waking sample and the 30 minutes postwaking sample. These procedures were repeated across all 3 collection days.

##### Sample Storage

After each sample was collected, participants were instructed to place the vial in their home freezer until study staff retrieved the samples.

### Sample Retrieval and Field Procedures

After completing the 3-day saliva collection period, study staff retrieved the frozen samples, saliva collection logs, and study phones from the participants’ homes. Study staff also administered a brief survey assessing participants’ understanding and adherence to the saliva collection protocol and reporting the study-issued phone condition (Multimedia Appendix 2). Participants received compensation (US $35) upon sample return.

Before the saliva retrieval home visit, study staff confirmed appointment times and assessed potential safety considerations. When safety concerns were present, alternative sample retrieval locations were arranged. Study staff documented any safety concerns in participant files, including a codeword that the participants could use to alert staff that they were unsafe and should call 911. Moreover, 2 study staff were present to conduct the retrieval visits, typically conducted on weekdays during daytime hours. Exceptions were made on a case-by-case basis for saliva sample retrieval visits during the weekend or at a location other than the participant’s home (ie, their workplace or a friend’s home).

At retrieval, study staff visually inspected each vial to verify the presence of at least 1 mL frozen saliva and recorded vial ID numbers, saliva collection information, and retrieval visit details. Study staff also administered a brief survey assessing participants’ understanding and adherence to the saliva collection protocol. Samples were then transported in a small cooler with ice packs to a laboratory deep freezer at Johns Hopkins University, where they were stored at −80 °C until shipped out for assay.

### Trauma-Informed Protocol

The saliva self-collection protocol was developed using trauma-informed principles to enhance feasibility, acceptability, and adherence among women with histories of abuse. Standardized procedures emphasized safety, autonomy, and privacy by allowing self-collection at home. Study materials were scripted with nonstigmatizing, culturally sensitive language and included explicit statements of voluntary participation, permitting participants to pause or discontinue at any time. Although collection schedules were standardized, the protocol allowed for flexible collection schedules, and reminders were delivered using preapproved methods. Participants were also provided with community and psychosocial support resources. Collectively, these procedures were designed to minimize retraumatization while supporting protocol compliance.

### Field Coordination

A full-time field coordinator oversaw saliva collection procedures, maintained participant contact, and coordinated sample retrieval. The field coordinator was a Black woman who was a member of the local community, which facilitated trust and communication with participants and allowed her to understand the participants’ lived experiences. Participants responded positively to the field coordinator’s one-on-one supportive phone calls. This role was critical to providing support for adherence to the saliva collection protocol.

The field coordinator provided great insight into the facilitators and barriers to study participation and to saliva collection, helping to refine the saliva collection protocol in several instances. First, the protocol originally specified a collection schedule based on recruitment date (ie, those recruited into the study on a Monday would collect saliva on Tuesday through Thursday). The field coordinator reported that a predetermined saliva collection start schedule did not fit many participants’ life demands. The protocol was adjusted to allow for a flexible collection start date. Second, the field coordinator was flexible to changes in call or text times and saliva retrieval schedules, often working outside of typical business hours to meet participants’ unique needs and facilitate their study participation. This support and much-needed flexibility allowed participants to better adhere to the saliva collection protocol. Third, the field coordinator conducted in-home saliva collection demonstrations for participants who needed the extra support, adding further support for protocol adherence.

### Sample Selection for Analysis

After saliva samples were retrieved, the 2 collection days most adherent to the protocol were chosen for shipping, assaying, and analysis. We developed a decision tree algorithm (Multimedia Appendix 3) to identify the 2 most protocol-adherent collection day samples to be assayed. Protocol adherence was evaluated using time data from saliva logs, time-stamped photographs, and SMS text messages. The algorithm prioritized samples collected closest to the target timing (eg, immediately upon waking and 30-min post waking) and gave greater weights to samples in which collection time was only manually noted on the saliva log, and for every 5-minute delay in saliva collection. The 2 most adherent collection days (with the lowest total weight) were selected for assay.

### Sample Storage and Shipping

Saliva samples were frozen immediately after collection and kept frozen for analysis. Samples retrieved from participants in Baltimore, Maryland, were stored at –80 °C at Johns Hopkins University until shipment and then batch shipped overnight on dry ice in accordance with federal regulations for biological materials to the Institute of Interdisciplinary Salivary Bioscience Research at the University of California, Irvine.

### Cortisol Assay

At the Institute of Interdisciplinary Salivary Bioscience Research, on the day of assay, samples were thawed and centrifuged to remove mucins and other particulates. Cortisol concentrations were measured in duplicate using a commercially available enzyme immunoassay (Salimetrics). The assay had a lower detection limit of 0.007 μg/dL, a range up to 3.0 μg/dL, and average inter- and intra-assay coefficients of variation below 10% and 5%, respectively [36]. The average of duplicate assay results for each sample was used in the statistical analyses.

### Measures

*Outcome variables* included (1) completing the saliva collection protocol, (2) saliva collection log, (3) saliva collection protocol adherence weight, and (4) cortisol levels. *Completing the saliva collection protocol* included women who provided at least 1 saliva sample that was sent off for assay. *Saliva collection log* survey measured participants’ adherence, understanding, and experiences with the at-home saliva collection protocol. Items captured compliance with instructions (eg, reviewing logs and following precollection restrictions), the usefulness of study reminders (eg, SMS text messages), assessed challenges (eg, remembering collection times and storing samples), as well as documented field notes. *Saliva collection protocol adherence weight* was a weighted score derived from the decision tree algorithm. For each collection day, weights (range 6‐14 points) were assigned to each of the 2 samples collected (ie, waking sample and 30-min postwaking sample) based on the method of time verification (eg, time-stamped photo or text vs manual log) and adherence to the protocol time sampling windows, with penalties applied in 5-minute increments for delays. The 2 weights were summed for a total daily sample adherence weight (range 12‐28 points). Lower scores indicated greater adherence to the protocol. *Cortisol levels* were reported as waking cortisol levels (μg/dL), 30-minute postwaking cortisol levels (μg/dL), and CAR. CAR was calculated by the area under the curve between cortisol measures upon waking and 30 minutes post waking using the following equation: CAR=((Cortisol 30-min post-waking+cortisol waking)/2)×T1)–(cortisol waking×T1), where T1 represents time elapsed between sample collections.

*Other variables* included (1) adult forced sex exposure and (2) sociodemographic variables. *Adult forced sex exposure* status was measured as a positive response to one of the following questions: (1) “Since you turned 18, has a male sex partner: used threats to make you have sex when you did not want to or used force (like hitting, holding down, or using a weapon) to make you have sex?” and (2) “Since you turned 18, has any other male done any of the following: used threats to make you have sex when you did not want to or used force (like hitting, holding down, or using a weapon) to make you have sex?” *Socio-demographic variables* included age, education, employment status, housing instability, annual individual income, relationship status, number of children, and sexual orientation.

### Statistical Analysis

All statistical analyses were conducted using Stata (version 15; StataCorp) [37]. Of the 305 women who participated in the study with complete survey data, 228 women completed saliva collection, with 191 (84%) women completing at least 1 full day of saliva collection that fell within acceptable range of time collection for CAR measurement (eg, waking sample collected within 15 min of wake time, 30-min postwaking sample collected between 30‐45 min postwake time, and ≥20 min time difference between collection of waking and postwaking samples; data not shown).

We began our analysis with descriptive statistics of the sociodemographic characteristics of women in the overall sample who completed the saliva collection protocol. Chi-square statistic was used for categorical variables, and the *F* statistic from ANOVA was used for continuous variables to detect statistically significant differences by adult sexual violence exposure, using *P*<.05 for statistical significance cutoff. We then described the frequency distribution of responses to the saliva collection log, which gave insight into the feasibility and use of supports for adherence to the saliva collection protocol, and compared differences by adult sexual violence exposure. We then described the protocol adherence feasibility and reported the average sample adherence weights for the total sample and by adult sexual violence exposure. Finally, we reported the salivary cortisol levels for the total sample and by adult sexual violence exposure for the waking and 30-minute postwaking samples, and the CAR for the subsample of women who completed at least 2 full days of saliva collection within acceptable time ranges. We used the Pearson correlation coefficient to assess the significant correlation of cortisol levels between collection days.

## Results

### Overview

Overall, our results report on our saliva collection protocol results, and demonstrate the feasibility of saliva CAR self-collection among Black women with histories of abuse. Of the 305 women who were eligible and consented to the survey and saliva collection, 228 (75%) completed the saliva specimen collection protocol, 33 (11%) were lost at the survey, 23 (8%) refused the saliva collection, and 21 (7%) did not complete the saliva collection (refused mid-completion; data not shown). Of the women who completed the saliva specimen collection protocol, 93 (41%) had a history of forced sex since age 18 years (exposed women) and 135 (59%) had no history of forced sex since age 18 years (unexposed women).

Table 1 describes the demographic characteristics of the total study sample that completed the saliva collection protocol and compares differences by adult forced sex exposure. Among the total sample of Black women who completed the saliva collection protocol (n=228), participants were on average 26.6 (SD 6.45) years old, most had a high-school education or more (190/228, 83%), and over half were currently employed (133/228, 58%). Socioeconomic vulnerability was evident, with 61% (139/228) reporting annual incomes below US $10,000 and 7% (16/228) reporting housing instability. Most participants were single (160/228, 70%), had at least 1 child (118/228, 52%), and identified as heterosexual (192/228, 84%). Women exposed to adult sexual violence who completed the saliva collection were, on average, significantly older, were less likely to have a high-school education, were less likely to be currently employed, and were more likely to report housing instability than women unexposed to adult sexual violence who completed the saliva collection.

**Table 1.** Sociodemographic characteristics of Black women with violence experience completing repeated measures self-collection of saliva in Baltimore, Maryland (Source: The ESSENCE Project 2015-2018).

Characteristic	Total saliva (n=228), n (%)	Adult forced sex exposure status		
		Exposed (n=93)	Unexposed (n=135)	*P* value^a^
Age^a^ (y), mean (SD)	26.61 (6.45)	28.75 (0.73)	25.13 (0.48)	<.001
18‐24, n (%)	96 (42)	26 (28)	70 (52)	
25‐34, n (%)	102 (45)	47 (51)	55 (41)	
35‐44, n (%)	30 (13)	20 (22)	10 (7)	
High-school education or more, n (%)	190 (83)	72 (77)	118 (87)	.047
Currently employed, n (%)	133 (58)	45 (48)	88 (85)	.01
Housing instability, n (%)	16 (7)	13 (14)	3 (2)	.001
Annual individual income (US $), n (%)				.27
<10,000	139 (61)	62 (67)	77 (57)	
10,000‐29,999	71 (31)	26 (28)	45 (33)	
≥30,000	18 (8)	5 (5)	13 (10)	
Relationship status, n (%)				.40
Single	160 (70)	63 (68)	97 (72)	
In relationship	62 (27)	26 (28)	36 (27)	
Separated, divorced, widowed, or other	6 (3)	4 (4)	2 (1)	
Number of children^a^, mean (SD)	1.07 (1.32)	1.12 (0.14)	1.04 (0.11)	.68
0, n (%)	110 (48)	44 (47)	66 (49)	.91
1 to 2, n (%)	84 (37)	34 (37)	50 (37)	—^b^
3 or more, n (%)	34 (15)	15 (16)	19 (14)	—
Sexual orientation, n (%)				.007
Heterosexual	192 (84)	71 (76)	121 (90)	
Bisexual or gay	36 (16)	22 (24)	14 (10)	

a *P* value for statistical difference by adult forced sex exposure status using chi-square for comparisons among categorical variables and the *F* statistic for comparison of means among continuous variables.

b Not applicable.

### Saliva Collection Logistics

Analysis of the saliva collection log data (Table 2) demonstrates high levels of protocol feasibility, regardless of adult forced sex exposure. In terms of the saliva collection demo, most women reported receiving the saliva demo in the clinic 192/228, 84%), most reported that the saliva demo at the clinic was thorough enough to remember how to self-collect (190/228, 83%), and 99% (225/228) returned the saliva log, which was the standard protocol. In terms of protocol adherence supports, most women reported referring to the instructions page provided to help remember how to collect the saliva sample (160/228, 70%). The most difficult part of the saliva collection reported was drooling (74/228, 33%) followed by taking pictures of the sample (46/228, 20%). Most women reported having a little or moderate difficulty refraining from drinking, eating, smoking, or brushing their teeth during the saliva collection (125/228, 55%). In terms of the study phones, most women reported receiving texts from the study phone (206/228, 90%), with most reporting that these texts were moderately useful (131/228, 58%) or very useful (56/228, 25%), and most using the study phone texts to wake up to collect their first sample (140/228, 61%). The majority of women reported taking pictures of the saliva vials (211/228, 93%). In terms of saliva storage, most women reported no problems storing the saliva samples in their freezer (217/228, 95%). There were no statistically significant differences observed between women exposed and unexposed to adult forced sex across the saliva collection log measures, indicating that the protocol was feasible across exposure groups.

**Table 2.** Saliva self-collection logistics among Black women with violence experience, by adult forced sex exposure status (n=228), Baltimore, Maryland (Source: The ESSENCE Project 2015-2018).

Characteristic	Saliva total (n=228), n (%)	Adult forced sex exposure status		
		Exposed (n=93), n (%)	Unexposed (n=135), n (%)	*P* value^a^
Saliva demo				
Saliva demo location				—^b^
In clinic	192 (84)	76 (82)	116 (86)	
In home	1 (0)	1 (1)	0 (0)	
In office	30 (13)	13 (14)	17 (13)	
Missing	5 (2)	3 (3)	2 (1)	
Saliva demo at clinic was thorough enough to remember how to collect				.09
Yes	190 (83)	72 (77)	118 (87)	
No	26 (11)	13 (14)	13 (10)	
Missing	12 (5)	8 (9)	4 (3)	
Saliva log				
Returned the saliva log				.36
Yes	225 (99)	91 (98)	134 (99)	
No	3 (1)	2 (2)	1 (1)	
Protocol adherence supports				
Referred to saliva instructions page to help remember how to collect				.22
Yes	160 (70)	71 (76)	89 (66)	
No	57 (25)	19 (20)	38 (28)	
Missing	11 (5)	3 (3)	8 (6)	
Most difficult part of saliva collection				.42
Drooling	74 (32)	35 (38)	39 (29)	
Taking pictures	46 (20)	19 (20)	27 (20)	
Remember when to collect sample	30 (13)	12 (13)	18 (13)	
Replying to texts	20 (9)	8 (9)	12 (9)	
Logging info	9 (4)	1 (1)	8 (6)	
Missing	49 (21)	18 (19)	31 (23)	
Difficult to refrain from drinking, eating, smoking, or brushing teeth				.20
Not at all difficult	54 (24)	16 (17)	38 (28)	
A little or moderately difficult	125 (55)	52 (56)	73 (54)	
Difficult	27 (12)	15 (16)	12 (9)	
Very difficult	6 (3)	2 (2)	4 (3)	
Missing	16 (7)	8 (9)	8 (6)	
Study phones				
Received texts from study phone				.61
Yes	206 (90)	86 (92)	120 (89)	
No	17 (7)	5 (5)	12 (9)	
Missing	5 (2)	2 (2)	3 (2)	
Study phone texts were very useful in helping me collect saliva				.75
Not at all useful	38 (17)	13 (14)	25 (19)	
Moderately useful	131 (57)	57 (61)	74 (55)	
Very useful	56 (25)	22 (24)	34 (25)	
Missing	3 (1)	1 (1)	2 (1)	
Used study phone texts to wake up to collect your sample				.16
Yes	140 (61)	64 (69)	76 (56)	
No	73 (32)	24 (26)	49 (36)	
Missing	15 (7)	5 (5)	10 (7)	
Took pictures of the saliva vials				.50
Yes	211 (93)	84 (90)	127 (94)	
No	14 (6)	7 (8)	7 (5)	
Missing	3 (1)	2 (2)	1 (1)	
Saliva storage				
Any problems storing the samples in the freezer				.24
Yes	8 (4)	1 (1)	7 (5)	
No	217 (95)	91 (98)	126 (93)	
Missing	3 (1)	1 (1)	2 (1)	

a *P* value for statistical difference between adult forced sex exposure status using *χ*2 for comparisons.

b Not applicable.

Table 3 describes the feasibility of protocol completion and assesses protocol adherence using the protocol adherence weights generated by the decision tree algorithm. Protocol completion was highly feasible, with the majority (191/228, 84%) of the total sample successfully completing at least 1 full day of saliva collection within the specific timing necessary to measure CAR. Of the total sample, 26% (59/191) achieved 1 full day of adherence to protocol collection, and 58% (132/191) achieved 2 full days of adherence. This was consistent across adult forced sex exposure categories, with 83% (77/93) of exposed women and 84% (114/135) of unexposed women completing at least 1 full day of adherent saliva collection, with no significant differences. In terms of protocol adherence weights, the total sample maintained an excellent mean adherence weight of 16.25 (SD 6.303). There were statistically significant differences in the total protocol adherence weights by adult forced sex exposure status, with exposed women demonstrating significantly less adherence to the protocol (mean 17.85, SD 5.217) than unexposed women (mean 15.16, SD 6.750; *P*=.002). This discrepancy was most pronounced among women who completed the full 2-day protocol, where exposed women remained significantly less adherent to the protocol than their unexposed counterparts (mean 17.36, SD 4.986 vs mean 14.48, SD 6.126, respectively; *P*=.004). This finding suggests that, although feasibility was high across both groups, trauma history may influence women’s ability to adhere to the protocol.

**Table 3.** Compliance to saliva self-collection protocol using adherence weights for Black women with violence experience completing repeated measures, by adult forced sex exposure status, Baltimore, Maryland (Source: The ESSENCE Project 2015-2018).

Protocol feasibility	Saliva total		Adult forced sex exposure status				
	n (%)	Mean (SD)	Exposed		Unexposed		*P* value^a^
			n (%)	Mean (SD)	n (%)	Mean (SD)	
Total samples collected within time range	228 (100)	—^b^	93 (100)	—	135 (100)	—	.88
0 full day	37 (16)	—	16 (17)	—	21 (16)	—	
1 full day	59 (26)	—	25 (27)	—	34 (25)	—	
2 full days	132 (58)	—	52 (56)	—	80 (59)	—	
Sample adherence weight							
Total sample	191 (100)	16.25 (6.303)	77 (100)	17.851 (5.217)	114 (100)	15.164 (6.750)	.002
1 full day	59 (31)	17.669 (7.048)	25 (32)	18.880 (5.632)	34 (30)	16.779 (7.894)	.24
2 full days	132 (69)	15.612 (5.857)	52 (68)	17.356 (4.986)	80 (70)	14.478 (6.126)	.004

a Of samples with adherence weights; *P* value for statistical difference between Exposed/Unexposed using Chi-square for comparisons among categorical variables & F-statistic for comparison of means among continuous variables.

b Not applicable.

Table 4 reports the cortisol levels among women who completed 2 full days of saliva collection within the required time limits for CAR, comparing the Pearson correlation coefficient of between-day cortisol levels (waking, post waking, and CAR) for each of the 2 days. Among the total sample, day 1 and day 2 cortisol waking samples (*r*=0.276; *P*=.001) and the CAR levels (*r*=0.178; *P*=.04) were weakly but statistically significantly correlated, whereas the postwaking samples (*r*=0.411, *P*<.001) were moderately and statistically significantly correlated. When stratifying by adult forced sex exposure, waking cortisol samples for day 1 and day 2 were weakly but statistically significantly correlated among exposed women (*r*=0.316, *P*=.02) and unexposed women (*r*=0.258, *P*=.02). Postwaking cortisol samples for day 1 and day 2 were moderately and significantly correlated among exposed women (*r*=0.505, *P*<.001) and unexposed women (*r*=0.380, *P*=.001). CAR levels for day 1 and day 2 were not statistically correlated among exposed women (*r*=0.027, *P*=.85); however, CAR levels were weakly but statistically significantly correlated among unexposed women (*r*=0.273, *P*=.01). Overall, postwaking cortisol demonstrated the greatest reproducibility across the 2 days, while CAR demonstrated the least stability, particularly among women with adult forced sex exposure.

**Table 4.** Salivary cortisol levels for Black women with violence experience completing 2 days of adherent saliva self-collection, by adult forced sex exposure status, Baltimore, Maryland (Source: The ESSENCE Project 2015-2018).

	Total		Adult forced sex exposure status				*P* value^a^	
			Exposed		Unexposed		Sample 1	Sample 2
	Sample 1	Sample 2	Sample 1	Sample 2	Sample 1	Sample 2
Waking^b^							.75	.44
Women, n	132	132	52	52	80	80		
Mean (SD)	0.29 (0.26)	0.24 (0.17)	0.30 (0.25)	0.25 (0.23)	0.29 (0.26)	0.23 (0.13)		
Median (IQR)	0.21 (0.131 to 0.366)	0.21 (0.125 to 0.289)	0.21 (0.126 to 0.415)	0.21 (0.131 to 0.280)	0.21 (0.134 to 0.362)	0.22 (0.124 to 0.308)		
Post waking^c^							.82	.55
Women, n	132	—^d^	52	—	80	—		
Mean (SD)	0.37 (0.30)	0.34 (0.21)	0.36 (0.25)	0.35 (0.17)	0.37 (0.33)	0.33 (0.23)		
Median (IQR)	0.30 (0.175 to 0.296)	0.32 (0.168 to 0.457)	0.29 (0.199 to 0.517)	0.35 (0.218 to 0.463)	0.31 (0.173 to 0.514)	0.28 (0.165 to 0.433)		
CAR^e,f^							.42	.68
Women, n	132	—	52	—	80	—		
Mean (SD)	1.287 (3.20)	1.637 (2.95)	1.007 (3.15)	1.504 (3.01)	1.469 (3.24)	1.723 (2.93)		
Median (IQR)	0.822 (−0.405 to 2.738)	1.137 (0.0675 to 2.948)	0.750 (−0.473 to 2.675)	1.419 (0.030 to 3.109)	0.867 (−0.402 to 2.813)	0.780 (0.122 to 2.666)		

a *P* value for statistical difference between exposed or unexposed using *F* statistic for comparison of means among continuous variables.

b Total: *r*=0.276, *P*=.001; exposed: *r*=0.316, *P*=.02; unexposed: *r*=0.258, *P*=.02.

c Total: *r*=0.411, *P*<.001; exposed: *r*=0.505, *P*<.001; unexposed: *r*=0.380, *P*=.001.

d Not applicable.

e CAR﻿: cortisol awakening response.

f Total: *r*=0.178, *P*=.04; exposed: *r*=0.027, *P*=.85; unexposed: *r*=0.273, *P*=.01.

## Discussion

### Principal Findings

The study developed a standardized, trauma-informed protocol for multiple day at-home salivary CAR self-collection for women with histories of abuse and provides evidence supporting the overall feasibility of implementing the protocol among a hard-to-reach sample of US Black women with experiences of trauma and abuse.

Overall participants demonstrated high engagement with study procedures, with the majority returning saliva logs, using adherence supports (ie, SMS text message reminders and referring to instructional materials), and adhering to key protocol components (ie, documenting sample collection times and freezing samples). However, findings highlight critical differences in protocol feasibility and adherence among women who completed the full 2-day collection by adult sexual violence exposure status, with exposed women demonstrating lower protocol adherence scores than unexposed women. Future protocols may benefit from enhanced trauma-informed supports, such as more frequent reminders, simplified procedures, or real-time monitoring, to mitigate adherence challenges among this population.

The cortisol analyses provide additional insight into the reliability of the at-home self-collected salivary biomarkers. Postwaking cortisol levels demonstrated the strongest reproducibility across days, while CAR demonstrated weaker and less consistent correlations, especially among women exposed to adult forced sex. The lack of significant day-to-day correlation in CAR among exposed women may reflect both biological variability with trauma-related HPA axis dysregulation and the observed challenges with protocol adherence. These findings suggest that while home-based saliva self-collection for CAR measurement is a viable and inclusive approach for studying stress physiology in this population, careful attention to protocol adherence and collection day flexibility is warranted.

This study is innovative in developing a saliva collection protocol tailored for this underrepresented population. Best practices include the following: (1) the use of text reminders via cell phones to enhance participants’ adherence to the saliva collection protocol; (2) the use of study staff who were representative of the community to retrieve saliva samples; and (3) the creation of a saliva sample decision tree, which automated the decision of which 2 of 3 saliva samples were most adherent in such a large sample. Research implications include the facilitation of the collection of salivary biomarkers (including stress and inflammation measures) to promote research that examines the physiological and health repercussions of GBV within the context of women’s everyday lives.

### Strengths and Limitations

The protocol had several strengths. First, we established an at-home saliva self-collection (and storage) protocol with accommodations for women currently experiencing violence. Second, we had a large sample size of Black women with violence experience to demonstrate feasibility and acceptability in a diverse sample with safety considerations. Our protocol, importantly, demonstrated feasibility and acceptability in 2 populations (Black women and Black women with abuse histories) that have classically been mistrustful of the health care system and health care research. Third, we explored the use of study-issued cell phones to improve protocol adherence in terms of timing of saliva collection (as CAR is time-sensitive) and feasibility of sending a time-stamped picture documenting the time of the collected sample. Finally, collecting saliva at-home captures the interaction between biological and behavioral processes in the context of participants’ everyday life, potentially enhancing the information collected compared with collecting biological markers in clinical settings.

Our protocol had several limitations that researchers implementing similar studies may wish to address. First, documenting the timing of saliva collection using the standard written log may have introduced self-report bias. To mitigate this, participants were instructed to send a time-stamped photo immediately after collecting each saliva sample. However, adherence to this protocol procedure was inconsistent due to logistical barriers (eg, uncharged or inaccessible study phone). Future studies could improve data accuracy by implementing automated time-stamping technologies (eg, mobile apps and electronic monitoring devices). Second, establishing regular wake times for participants for 3 consecutive days was challenging, particularly for participants who worked nights or with irregular sleep schedules. Although participants were provided with standardized instructions to plan sample collection based on their primary wake time, wake time ambiguity persisted among participants with fragmented sleep patterns. This variability may have introduced “noise” in terms of timing of sample collection relative to the cortisol diurnal pattern. Future research should consider more flexible, individualized collection protocols for women with abuse histories. Third, study-issued phones were intended to reduce the associated cost and participant burden of using their personal phone. However, some participants did not consistently use the study-issued phones, misused them for personal purposes, or reported loss or damage. Most participants had their own cell phone, and it was difficult for them to carry and use 2 cell phones. Additionally, for participants with histories of abuse, carrying a secondary phone may have posed safety concerns (ie, raising the suspicions of an abuser), which potentially affected protocol adherence. Future studies should carefully weigh the risks and benefits of providing study-specific devices and consider alternatives (eg, secure mobile app compatible with personal phones). Fourth, although access to a refrigerator was an eligibility requirement, participants who lived in settings with a shared refrigerator (ie, homeless or domestic violence shelter) had their samples erroneously thrown out. Other participants reported that family members found the samples and, not knowing what they were, threw them out. In these instances, staff issued new supplies. To mitigate this risk, future protocols should include more comprehensive participant education regarding sample storage, as well as considerations for discreet labeling. Finally, the use of cryovials with color-coded caps to visually distinguish waking sample (green) and 30-minute postwaking sample (red) vials may constitute a study limitation for individuals with red-green vision deficits. Future research should consider adopting alternative visual markers (eg, distinct patterns, textual labels, or uniquely shaped indicators) to enhance accessibility and ensure clarity for all participants.

### Future Directions

Future studies that seek to obtain salivary samples among racial and ethnic minority women with histories of abuse should consider the context in which the women are living. In our study, we incorporated safe words and established safety plans with participants during key points of the protocol (ie, sample collection, phone usage, and sample pick-up). In addition, we preprogrammed community resources into the study phone contacts (ie, domestic violence shelters and study contacts) under aliases.

Acknowledging the reality and potential volatility of the context of participants’ lives signals support for them and builds trust, which can strengthen protocol adherence. By strengthening adherence, we can advance research that examines the physiological and health repercussions of GBV.

## Supplementary material

10.2196/64150 Multimedia Appendix 1 Saliva collection log.

10.2196/64150 Multimedia Appendix 2 Saliva retrieval form.

10.2196/64150 Multimedia Appendix 3 Saliva decision tree.

## Abbreviations

CAR: cortisol awakening response
ESSENCE: Examining Stress, Sexual Experiences, and Neighborhood Correlates of HIV Risk among Black Women
GBV: gender-based violence
HPA: hypothalamic-pituitary-adrenal
IPV: intimate partner violence
STD: sexually transmitted disease
