# Supplementary material for: Repeat At-Home Saliva Collection to Measure Cortisol Awakening Response (CAR): Protocol Feasibility Study Among Black Women With Histories of Abuse
Source: Interact J Med Res. 2026 Sep 3;15:e64150. doi: 10.2196/64150 (PMC13541225; doi:10.2196/64150)
Supplement: Supplementary file 1 [file ijmr-v15-e64150-s001.docx]

**APPENDIX 1: Saliva Collection Log**
